# The Early Treatment of Certain Diseases

**Published:** 1913-10

**Authors:** A. W. Stirling

**Affiliations:** M. D., C. M. (Edin.), D. P. H. (Eng.); Opthalmic Surgeon to Grady Hospital, etc.; Atlanta, Ga.


### Journal-Record of Medicine

Successor to Atlanta Medical and Surgical Journal, Established 1855
and Southern Medical Record, Established 1870

**OWNED BY THE ATLANTA MEDICAL JOURNAL COMPANY**

PUBLISHED MONTHLY

*Official Organ Fulton County Medical Society, State Examining Board, Presbyterian Hospital, Atlanta, Birmingham and Atlantic Railroad Surgeons' Association, Chattahoochee Valley Medical and Surgical Association, Etc.*

EDGAR BALLENGER, M. D., Editor
BERNARD WOLFF, M. D., Supervising Editor
A. W. STIRLING, M. D., C. M., D. P. H.; J. S. HURT, B. Ph.. M.D.
GEO. M. NILES, M. D., W. J. LOVE, M. D., (Ala.); Associate Editors
E. W. ALLEN, Business Manager

**COLLABORATORS**

DR. W. F. WESTMORLAND, General Surgery
F. W. McRAE, M. D., Abdominal Surgery
H. F. HARRIS, M. D., Pathology and Bacteriology
E. B. BLOCK, M. D., Diseases of the Nervous System
MICHAEL HOKE, M. D., Orthopedic Surgery
CYRUS W. STRICKLER, M. D., Legal Medicine and Medical Legislation
E. C. DAVIS, A. B., M. D., Obstetrics
E. G. JONES, A. B., M. D., Gynecology
R. T. DORSEY, JR., B. S., M. D., Medicine
L. M. GAINES, A. B., M. D., Internal Medicine
GEO. C. MIZELL, M. D., Diseases of the Stomach and Intestines
L. B. CLARKE, M. D., Pediatrics
EDGAR PAULIN, M. D., Opsonic Medicine
THEODORE TOEPEL, M. D., Mechano Therapy
R. R. DALY, M. D., Medical Society
A. W. STIRLING, M. D., Etc., Diseases of the Eye, Ear, Nose and Throat
BERNARD WOLFF, M. D., Diseases of the Skin
E. G. BALLENGER, M. D., Diseases of the Genito-Urinary Organs

VOL. LX. ATLANTA, GA., OCTOBER, 1913. NO. 7

### *THE EARLY TREATMENT OF CERTAIN DISEASES

BY A. W. STIRLING, M. D., C. M., (EDIN.), D. P. H. (ENG.)

OPHTHALMIC SURGEON TO GRADY HOSPITAL, ETC.

ATLANTA, GA.

It is so long since I sent in the title of this little paper that, when the president a few days ago mailed me notice that it was nearly due, I admit that I had forgotten all about it. I can barely remember why I chose it, but I think I recall that is was because at the moment, as frequently before and since, I was considerably exercised by the sight of patients unnecessarily suffering on account of the procrastination of themselves or their medical advisors. It does not tend to elevate one's respect for one's own profession when in these days of comparative enlightment, the patient explains the tardiness of his appearance for treatment by the assertion that the doctor said he would "grow out of it". Out of how many troubles, besides, lying, teething and bed wetting, will a child naturally sometimes grow scathless? And how often even these may be mitigated by appropriate treatment applied to the proper situation? We may safely advise that the "out growing" process should be wiped from the medical vocabulary. It has resulted in much chronic invalidism, much acute suffering, many an unnecessary operation, and a distinct lowering of the general level of national health, as has also the natural tendency to postpone special treatment when special knowledge is necessary. I have time at the late date at which I am able to apply myself to this paper to say but a few words upon a few special diseases, and these addressed more to the general practitioner than to the specialist, my object being, out of the fullness of a sometimes bitter experience, to remind such as have not given any careful heed to the matter of the delicacy of the tissues involved in some of the few lesions concerning which I shall speak, and the consequent necessity of saving these as rapidly and as completely as possible from attack, and that in all serious consequences are not an improbable result of delay.

For instance, let us consider for a moment that most common of complaints—earache. Earache means congestion of the middle ear, and seems to be treated as a routine by the instillation of sweet oil. But the earache is usually secondary to some nasal inflammation, and if the nasal congestion continues so probably will the aural, and the aural may even begin and develop while the nasal is declining. If the aural inflammation progresses, serum will be thrown out, the drum head may be gradually or rapidly thinned till it bursts, when we have a running ear. Many doctors, as well as a large proportion of the laity think little or nothing of discharging ears, but they are in reality frequently a step nearer a mastoid operation, or at least chronic deafness, as well as much discomfort and trouble for child and parents. An inflamed middle ear should be treated as a serious matter, for a serious matter it may very soon become. Rapid and active interference are essential purgatives, bed, light diet, heat, possibly opiates and the frequent use, not of that excellent culture medium, sweet oil, but of preferably carbolic acid, cocain, and glycerine. Also it is better to open the drum head too soon than too late, because a clean incision will heal where the nectrotic edges of a perforation will not approximate.

The lives of too many have been seriously handicapped by chronic discharging ears (which also prohibit life insurance,) deafness and mutism from which early and thorough treatment would have saved them. When the acute symptoms have subsided, attention must be given to the removal of the cause which will generally be found to lie in the presence of adenoids (which may be large, or small, or of comparatively healthy, or of greatly diseased tissue). There may be diseased tonsils, though sometimes only an apparently uncomplicated coryza. To some of the other reasons for the early removal of adenoids, as from the point of view of resulting facial deformity, attention has frequently been called, more especially in recent years. I hardly think enough has been said, or that too much could be said, of the beneficient action of the operation upon the general health. I have been very forcibly struck by this. It is one of the noticeable triumphs of surgery. It is a safeguard in ralation to scarlet fever and diphtheria, and it is the only means to prevent the long train of symptoms which follow in the wake of the adenoids, and with which everyone is now becoming more or less familiar, and the child will not "grow out" of the disease. Many adenoid subjects reach adult life without an operation, and as they grow older appear not to suffer. But such of us as are constantly examining the vault of the pharynx see in these old adenoids the cause of a large proportion of their cases of so-called post-nasal catarrh, tinnitus, and deafness.

Diseased tonsils take a somewhat similar position in pathology. Their evil effects are not dependent so much, as a rule, upon mechanical obstruction as upon their unhealthiness, upon their being foci of disease for the surrounding tissues. Adenoids of course play a role of the same kind, but their obstructive feature is specially noticeable. Any part of the tonsillar ring may be the port of entry for a variety of diseases of which tubercle is specially to be remembered, and the question of the removal of tonsils is generally to be decided more by their pathological state than by their mere size. Small diseased tonsils with enlarged neck glands are more to be feared than a mere hypertropy which partially closes the lumen of the throat, if the nasal passages are free, a combination, however, which is rare before operation, because, in my experience, there are almost always adenoids where there are diseased tonsils. Diseased tonsils appear to be so commonly the route which dangerous affections take in their onslaught upon life that in our present state of knowledge he who willfully postpones their elimination when they are distinctly seriously affected cannot be considered to be doing his duty to those entrusted to his care.

In a somewhat different category, but capable of causing intense suffering, and also it may be said secondary distant pathological conditions, comes another affection which may usually be cured with little trouble during the first few days of its evil existence, but which, neglected on account of mistaken diagnosis or the expectation that it will soon pass of itself, often results in chronic pain and other almost equally disagreeable symptoms. I refer to commencing sinus suppuration, and more especially in the maxillary antrum.

How frequently is this mistaken for facial neuralgia, in reality a much rarer affection! and how seldom is it remembered that the pain is not concentrated over the position of the antrum, but rather referred to regions above or in the orbit! If a correct diagnosis is made, and if, instead of using the hypodermic needle or other pain concealers, it is borne in mind that the anatomy of the sinus was physiologically appropriate for a lower grade of animal development before the head was held so high as mankind holds it, and that for him, the only chance for drainage is a return to a more lowly disposition for say 48 hours, such cases in my experience will nearly always rise up on the way to health if not already there.

The patient is to lie on his face, head somewhat hanging, the affected side the higher, and to retain this position for 24 or 48 hours or even longer. And it need not be specially uncomfortable, nor the time hang heavily, because with a little ingenuity he can enjoy a good novel fairly oblivious to place and circumstance. To this inducement to free drainage may be added some such spray as will tend to contract the nasal mucous membrane, an alkaline wash to clear out the nose, and what general treatment may appear to be indicated. But the main hope of success lies in EARLY treatment. The postural treatment is worth trying even after the trouble has existed for two or three weeks, but the earlier it is begun the better the prospect.

I promised you a short paper, and therefore, of ocular diseases I shall mention only one, iritis. But to postpone its treatment seems to be such an every day occurrence that personally it would appear as if I were seldom without at least one case which had been seriously spoiled by initial delay. I believe I am right in saying that there is no disease whatsoever the results of treatment in which differ more in accordance with the rapidity in which active measures are instituted with the view of securing rest and the best physiological position for cure. If one will for a moment recall certain features of the ocular anatomy it will be apparent that early treatment of iritis is essential, and why. It will be remembered that the normal iris moves to and fro upon, or close to, the surface of the lens capsule, and that on account of the shape of the lens the more dilated the pupil, the farther say the iris recedes from the capsule. It will also not be forgotten that the iris is richly supplied with arteries running in a direct line from the periphery to the pupillary edge, and that it is the filling of these vessels which causes the contracted pupil in commencing iritis.

The objects of treatment are thus at once apparent. We wish to remove the iris as rapidly as possible from the lens before the gluey lymph thrown out by the inflamed iris binds iris and lens capsule together; and in order to do this we use a drug which will paralyze the nerve which produces contraction of the pupil and another which will stimulate the nerve which causes its dilation as well as contract the dilated arteries already referred to. The moment these effects have been obtained by a mydriatic like atropine combined with cocain the pain and redness diminish and the eye is already on the road to recovery. Internal medication is always indicated, and often leeching is invaluable where time has been lost and the result in doubt.

When the iris cannot be induced to leave the lens, the case is certain to be long drawn out, and the ultimate state of the eye can never be counted upon. Even if the inflammatory symptoms subside, the probable vitreous and aqueous opacities disappear, and apparently healthy sate supervene, that iris is yet liable to recurrent attacks which on account of its adhesions are unlikely to be easily cured, so much so that an iridectomy is frequently advisable with a view to preventing a gradual decline of the eye into a painful uselessness. No disease better than iritis points the moral "a stitch in time saves nine."

It would be out of place here to discuss the causes of iritis, but it is quite apropos to say a word or two in conclusion in urging such as may not be in the habit of examining the teeth of their patients to pay particular attention to them whenever faced by an obscure ailment, which might result from the absorption of poisons formed within the body. Not only is iritis undoubtedly not infrequently due to dental imperfections, and specially to pyorrhea, but it is practically certain than a number of other ocular affections, especially of the uveal tract may likewise take their rise in the teeth or gums. And if ocular affections, how much more likely diseases of parts more apparently connected with the mouth! Also, it is unnecessary to expect a general oral sepsis, for a single carious tooth or an abscess of one root may suffice to set up a severe and serious affection at a distance.

I should like as a final word to paraphrase the old adage, and to say relative to treatment, "Early to bed is early to rise".

